# Aging and Viral Respiratory Infections: Focus on Vitamin D and Microbiota

**DOI:** 10.1155/bmri/8581187

**Published:** 2026-09-09

**Authors:** Shahab Falahi, Amir Abdoli, Elham Aboualigalehdari, Ava Hashempour, Azra Kenarkoohi

**Affiliations:** ^1^ Zoonotic Diseases Research Center, Ilam University of Medical Sciences, Ilam, Iran, medilam.ac.ir; ^2^ Department of Parasitology, Faculty of Medical Sciences, Tarbiat Modares University, Tehran, Iran, modares.ac.ir; ^3^ Department of Parasitology and Mycology, Faculty of Paramedical Science, Ilam University of Medical Sciences, Ilam, Iran, medilam.ac.ir; ^4^ HIV/AIDS Research Center, Institute of Health, Shiraz University of Medical Sciences, Shiraz, Iran, sums.ac.ir; ^5^ Department of Laboratory Sciences, School of Allied Medical Sciences, Ilam University of Medical Sciences, Ilam, Iran, medilam.ac.ir

**Keywords:** aging, immune aging, microbiota, respiratory viral infections, vitamin D

## Abstract

Viral respiratory infections in older adults tend to be more severe and linked to increased mortality. Factors such as lung aging and changes in its structure and function; aging of the immune system and decrease in its function with age; and inflammaging, the accumulation of senescent cells with age with senescence‐associated secretory phenotype (SASP) (is a secretory profile that includes the secretion of inflammatory cytokines, growth factors, chemokines, and matrix‐metalloproteinases) leads to an increase in the susceptibility and vulnerability of older adults to respiratory infections. Malnutrition and micronutrient deficiencies, including rising rates of vitamin D deficiency, can also exacerbate respiratory infections in older adults. Vitamin D plays a role in maintaining epithelial integrity and has anti‐inflammatory properties. Vitamin D enhances innate immune responses and suppresses adaptive immune responses, thereby restoring the immune system to homeostasis. Vitamin D deficiency is associated with microbiota dysbiosis and inflammation, and higher vitamin D levels are associated with greater abundance of beneficial, protective microbiota against respiratory infections. A more profound comprehension of the advantageous impacts of vitamin D in respiratory viral infections, particularly the relationship between vitamin D and the microbiota, will provide new perspectives on the importance of vitamin D assessment and standardization.

## 1. Introduction

Viral respiratory infections remain a major public health problem and a leading cause of death, especially in the elderly [1]. Aging is associated with increased susceptibility to respiratory infections and more severe clinical outcomes [2]. Age‐related changes in host biology, such as altered lung structure and function, impaired immune regulation, and chronic inflammation, contribute to the increased vulnerability of the elderly to respiratory infections [2]. As a result, the elderly exhibit more severe clinical manifestations and outcomes during viral respiratory infections such as influenza, COVID‐19, and RSV9 [1].

Several factors influence the severity and outcome of viral infections of the airways in older people. Among these factors, nutritional status and the composition of the microbial population were identified as determinants of immune system and respiratory health [3]. Vitamin D is a known immunomodulatory molecule [4]. Vitamin D affects the innate and adaptive immune systems [5]. It has anti‐inflammatory activity and is involved in the regulation of inflammatory responses [3]. Vitamin D also helps maintain the integrity of the epithelial barrier in the airways [4]. Vitamin D is involved in the expression of antimicrobial peptides [5]. As regards the microbial community, the role of the microbial community in respiratory maturation and respiratory tract health is also well known. Microbiota are known to be part of the structure of the mucous membranes of the body and play a protective role against viral respiratory infections [3]. Furthermore, the microbiota play a role in the development of the immune system and in immune system training [3].

There is increasing evidence that vitamin D and the microbiota are functionally interconnected and, in some cases, have synergistic roles [6]. Both vitamin D and the microbiota play a role in the integrity of epithelial barriers and the maintenance of immune homeostasis. Vitamin D influences the microbiota population through its effects on epithelial barriers, the production of antimicrobial peptides [5], and microbiota metabolites that can activate vitamin D receptor (VDR) signaling [5]. Aging is often associated with vitamin D deficiency and microbiota dysbiosis [6, 7], both of which are linked to inflammation [5, 8] and can synergistically impair antiviral immunity and exacerbate inflammation during viral infections. There is a substantial body of evidence supporting the independent roles of vitamin D and the microbiota in immune function and viral respiratory infections. However, their bidirectional interaction in the context of aging and viral respiratory infections has received less attention.

The purpose of this narrative review is to examine how changes in the vitamin D status and composition of the gut flora, as well as their interactions, affect the immune response, severity and outcome of respiratory viral infections in elderly adults. In addition, the clinical implications of these interactions for the prevention and treatment of respiratory viral infections in older people are discussed.

## 2. Methods

This narrative review examined how vitamin D and the microbiota influence the severity of viral respiratory infections in older adults. Databases including PubMed, Scopus, Web of Science, and Google Scholar were searched for eligible articles for this review up to July 2026 using the following keywords and Boolean operators: (“Viral respiratory infection” OR “Respiratory viruses” OR “Influenza” OR “COVID‐19” OR SARS‐CoV‐2 OR “Respiratory Syncytial Virus” OR “RSV”) AND (“Vitamin D” OR “calcitriol” OR “Vitamin D supplements” OR “25(OH)D” OR “1,25(OH)2 D”) AND (“Aging” OR “Elderly” OR “Older adults” OR “Geriatric”) AND (“Microbiota” OR “Microbiome” OR “Dysbiosis”) AND (“Immune aging” OR “Immunosenescence”).

Inclusion criteria focused on relevant English‐language full‐text articles, randomized controlled trials (RCTs), and prospective cohort studies that investigated the effects of vitamin D and the microbiota on the severity of respiratory infections in older adults. Exclusion criteria applied were studies in non‐English languages, articles submitted as preprints, letters to the editor, conference abstracts, and articles without full text.

Articles were screened in two stages of title and abstract review for relevance, followed by full‐text assessment. Also, the reference lists of the retrieved eligible articles were manually screened to identify additional relevant publications.

This review attempts to synthesize the available scientific evidence on the impact of vitamin D and the gut microbiota, and their relationship, on the severity and outcomes of viral respiratory infections in older adults.

## 3. The Aging Host

Biological aging is a key factor in increased susceptibility to infections, including viral respiratory infections, due to the decline in the function of various body systems, such as the immune system [1]. Factors such as the aging of the lungs and alterations in their structure and function, including reduced lung elasticity, diminished capacity for repair and regeneration, and decreased mucociliary clearance, result in a lowered ability of the lungs to combat infections [9] [Part B].

The aging of the immune system (immunosenescence) and its functional decline with age manifest in several aspects [7], including thymic atrophy, increased fat tissue in the bone marrow, fibrosis in lymph nodes, reduced quantity and size of germinal centers, fewer naive B and T cells [10], a rise in Tregs, diminished phagocytic capacity of macrophages, impaired responses to Type 1 interferons [11], an increase in the number of nonfunctioning memory cells [10, 12], and a higher incidence of chronic infections like CMV followed by T cell fatigue [13], all contributing to a reduced ability of the immune system to combat infectious agents [7].

In addition to immunosenescence, the presence of a low‐grade sterile inflammatory state (inflammaging) [14] negatively influences the immune response. The increase in the number of senescent cells with aging, with the SASP phenotype (senescence‐associated secretory phenotype) (accumulation of senescent cells in the lung and secretion of inflammatory factors and creation of an inflammatory environment in the lung) [15, 16], is another factor that contributes to the increased severity of respiratory diseases in older adults.

Immunosenescence also affects pulmonary immunity [12]: The function of alveolar macrophages in clearing pathogens and dead cells is reduced [10, 12]. The phagocytic activity of mucosal dendritic cells decreases, and the migration to the draining lymph nodes for priming adaptive immune responses is impaired [12, 17]. The phagocytic activity of neutrophils is also reduced, and neutrophils accumulate in large numbers in aged lungs, persist for long periods, and may lead to tissue damage [12, 16]. There are also immunosenescence effects on T and B cells. In addition, immunosenescence leads to delayed repair responses during lung involvement, delaying recovery from respiratory disease in older adults [14].

The increasing incidence of comorbidities, along with a decrease in average body temperature, is associated with aging and has increased the susceptibility and vulnerability of older adults to respiratory infections [11]. Many RNA viruses replicate poorly or not at all at elevated or feverish temperatures [18].

The composition and function of the microbiota change with age [19]. In older adults, the abundance of beneficial bacteria decreases, whereas the abundance of pathogens and bacteria with inflammatory properties increases [7]. For example, the abundance of Bifidobacterium and Acremancy decreases in older adults, whereas the abundance of Enterococcus and Streptococcus increases [19]. Also, the abundance of bacteria that produce short‐chain fatty acids (SCFAs) decreases with age. SCFAs are products of the fermentation of dietary fibers in the gut by microbiota and have anti‐inflammatory and immunomodulatory properties. In general, older adults frequently experience dysbiosis, or detrimental alterations in the microbiota population. Increased inflammation, poor immune homeostasis regulation, and a reduction in the synthesis of anti‐inflammatory metabolites are all linked to age‐related dysbiosis, which may make older adults more vulnerable to respiratory viral infections. In addition, the phenomenon of leaky gut has been reported in the elderly [7]. Increased intestinal permeability makes it possible for antigens and microbial products to pass through the intestinal–epithelial barrier and cause inflammatory reactions. Continuous exposure of the immune system to these microbial components may contribute to chronic immune activation and the development of inflammaging. Over time, these processes may disrupt immune homeostasis and impair immune function, ultimately influencing antiviral immune responses and increasing the severity of respiratory viral infections in older adults.

The lung microbiota in the elderly has not been investigated because it is challenging to sample the lower respiratory tract [12]. Nonetheless, several studies have compared the microbiota of the upper respiratory tract in younger and older individuals. Findings suggest that the composition and relative abundance of the upper respiratory tract microbiota differ between older and younger individuals [20]. The respiratory tract microbiota in older individuals appears to be less stable, which may be related to the susceptibility to respiratory diseases in the elderly [12]. In animal studies, in aged mice, the composition of the upper respiratory tract microbiota did not return to its initial state even several weeks after infection, indicating a decrease in the resilience of the microbiota during aging [21].

Taken together, changes in microbial communities in older adults, including changes in the composition and abundance of microbiota, reduction of beneficial bacteria, lower resilience, lower stability, and lower diversity, may affect immune responses and the severity of viral respiratory infections in the elderly.

**Figure 1:**
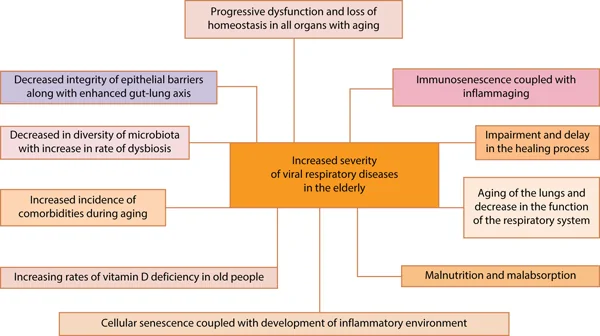
Several factors lead to an increase in the morbidity and mortality of viral respiratory diseases in older adults.

In addition to the aforementioned factors, nutrition and micronutrient status—particularly vitamin D levels—significantly influence the severity of respiratory infections [22, 23]. The risk of vitamin D deficiency increases significantly with age. This trend is exacerbated by factors such as malnutrition associated with aging, decreased sun exposure due to increased time spent indoors, and a decline in the skin′s ability to synthesize vitamin D, driven by lower concentrations of 7‐dehydrocholesterol in the skin [12]. Vitamin D deficiency can lead to reduced immune tolerance and abnormal immune responses [5]. All the mentioned factors that lead to an increase in the morbidity and mortality of viral respiratory diseases in older adults are shown in Figure 1.

## 4. Vitamin D as a Key Modulator

The precursors of vitamin D are vitamins D3 (cholecalciferol) and D2 (ergocalciferol) [24]. These precursors may be synthesized in the skin or obtained from the diet, and then converted in the liver by cytochrome P450 to 25‐hydroxyvitamin D (25(OH)D), its detectable and circulating form. In the kidney, 25(OH)D is converted to 1,25‐dihydroxy vitamin D (1,25(OH)2D) or calcitriol, the active form of vitamin D [24, 25]. Vitamin D (1,25(OH)2D) can bind to the VDR and lead to the activation of vitamin D signaling [4, 24]. Vitamin D–metabolizing enzymes, including CYP27B1 and the VDR, were also identified in lung epithelial cells and immune cells, supporting the association of vitamin D with respiratory infections, including viral infections [24].

Vitamin D has a complex function in the regulation of the immune system [26]. The VDR is present in most cells of the immune system [27]. This widespread expression indicates a direct effect of vitamin D on immune activity [28]. Vitamin D has anti‐inflammatory effects [8] by suppressing NF‐*κ*B, a critical mediator of the inflammatory process. Vitamin D controls the overproduction of proinflammatory cytokines and increases the release of anti‐inflammatory cytokines such as interleukin‐4, ‐5, and ‐10, as well as promoting the expansion of regulatory T cells [8, 28]. By reducing inflammation, vitamin D also limits the immunopathology in the response to an infection [29]. The activation of VDR also suppresses the differentiation of TH1 and TH17 cells, thereby further helping maintain immune homeostasis [30].

The innate immune system serves as the primary barrier against pathogens, including respiratory viruses [31]. Innate immunity utilizes pattern recognition receptors (PRRs) to identify common structures of pathogens, known as pathogen‐associated molecular patterns (PAMPs) [32]. Vitamin D boosts the innate immune system by elevating the expression of PRRs, improving pathogen detection [33]. Upon PRRS detection of a pathogen, immune cells initiate vitamin D metabolism. In fact, the metabolism of vitamin D in macrophages is linked to pathogen detection, which makes it a crucial component of the innate immune system. For instance, the binding of ligands to the TLR2/1 heterodimer in macrophages has been shown to enhance the expression of CYP27B1, an enzyme that catalyzes the conversion of 25(OH)D to 1,25(OH)2D [33].

The 1,25(OH)2D‐VDR complex binds to vitamin D response elements (VDREs) of the cathelicidin gene promoter to enhance cathelicidin transcription [34]. Beside its antimicrobial role, cathelicidin performs additional functions, including the induction of various proinflammatory cytokines, the stimulation of chemotaxis of neutrophils, monocytes, macrophages, and T cells to the site of infection, and the facilitation of respiratory pathogen clearance by triggering apoptosis and autophagy in infected epithelial cells [34]. The active form of cathelicidin is LL‐37 [27]. Antiviral properties of LL‐37 have been reported against several viral infections: LL‐37 can bind to the influenza virus and destroy its membrane [28], LL‐37 binds to the spike protein (S‐protein) and prevents the attachment of COVID‐19 to ACE2 and the entry of the virus, also by binding to viral dsRNA, LL‐37 facilitates dsRNA recognition by TLR3 and thus elicits antiviral responses [28].

Considering the extensive expression of TLRs across different cell types and their capacity to react to multiple pathogens, it is feasible that additional TLRs or PRRs enhance extrarenal‐renal expression of CYP27B1, and that 1,25(OH)2D generated locally in immune cells might exert wider influences on the immune response [33].

In the context of adaptive immunity, the activation of T‐ and B‐cells results in the upregulation of the VDR, which facilitates alterations in the expression of more than 500 genes related to vitamin D, emphasizing its comprehensive role in immune function [35].

Vitamin D has the immunomodulatory effects mentioned above, and therefore influences the immune response to viral respiratory infections. It enhances the innate immune system and balances adaptive immunity [36, 37]. In addition to its effects on the immune system, vitamin D influences the cellular antioxidant system [38]. Supplementing with vitamin D can influence the expression of genes associated with antioxidant responses and reduce oxidative stress–induced cellular damage in the context of respiratory infections [29]. Vitamin D plays a role in maintaining the integrity of the intestinal and lung epithelia [25, 29]: Vitamin D signaling increases the expression of tight junction proteins, strengthening the function of epithelial barriers [25, 30].

Viral respiratory infections represent a significant public health issue that imposes a substantial burden on healthcare systems [39]. These infections frequently correlate with more serious consequences in older adults [40]. In recent years, particularly during the COVID‐19 pandemic, greater focus has been placed on the importance of micronutrients in combating respiratory infections, such as vitamin D [40]. Several studies have demonstrated the potential protective benefits of vitamin D against viral respiratory infections [41, 42].

Respiratory syncytial virus (RSV) is typically associated with serious illness in young children and older adults, whereas in healthy individuals (adults and children), it typically causes a common cold [43]. A study has demonstrated the positive effects of vitamin D on RSV [44]. The study indicated that vitamin D deficiency in the cord blood of healthy infants was associated with a higher risk of RSV‐related lower respiratory tract infections (LRTI) during their first year. Regular vitamin D supplementation during pregnancy is suggested as an effective approach to prevent RSV‐related LRTI in infants [44].

In primary human tracheobronchial epithelial cells exposed to 1,25(OH)2D prior to RSV infection, the levels of the NF‐*κ*B inhibitor I*κ*B*α* were elevated—the rise in I*κ*B*α* levels results in the deactivation of NF‐*κ*B. Consequently, vitamin D inhibits the activation of NF‐*κ*B–driven secretion of IFN‐*β* and CXCL10 by RSV [33, 45]. Another study showed a connection between specific single‐nucleotide polymorphisms (SNPs) in the VDR and genetic vulnerability to RSV bronchiolitis [46]. Despite the aforementioned studies indicating potential protective benefits of vitamin D against RSV, further epidemiological research and clinical trials are required to explore the impact of vitamin D on RSV in older adults.

Influenza is a respiratory illness linked to significant morbidity and mortality each year and can lead to serious outcomes in populations like children, pregnant women, and older adults [47].

A study involving school‐age children has demonstrated that vitamin D3 supplementation in winter might reduce the incidence of influenza A [48].

The SARS‐CoV‐2 virus, which causes COVID‐19, is a respiratory virus that has caused a pandemic in recent years [49]. Age was a major factor in the increased severity of the disease, and older adults were among the groups at highest risk of COVID‐19 infection [50, 51]. Research findings indicated a connection between vitamin D deficiency and the severity of COVID‐19 infections [52]. The research suggested that vitamin D supplements be given to hospitalized COVID‐19 patients to improve clinical outcomes [52]. Findings from a cohort study of patients aged 65 and older with COVID‐19 indicated that those with vitamin D deficiency may experience poorer clinical outcomes [53]. A cross‐sectional study found that most hospitalized patients with COVID‐19 or influenza A had vitamin D deficiency, which was associated with the severity of the illness. These results underscore the need for further research to determine whether vitamin D deficiency contributes to the progression of the illness to a more severe stage [54].

In infections such as influenza and COVID‐19, dysregulated immune responses and heightened inflammation contribute to disease development and tissue injury [55, 56]; vitamin D has been suggested to help protect by reducing inflammation, thereby minimizing inflammation‐related tissue damage [4, 10, 28, 29]. Vitamin D may reduce the likelihood of a cytokine storm, which is linked to tissue damage in influenza and COVID‐19 [4, 55]. Vitamin D may decrease the synthesis of proinflammatory cytokines such as tumor necrosis factor *α* and interferon *γ* [29, 56]. Vitamin D supplementation lowers the levels of proinflammatory cytokines and boosts the levels of anti‐inflammatory cytokines produced by macrophages [29].

The relationship between vitamin D levels and the severity of respiratory viral infections is not yet fully understood. Although mechanistic studies suggest a protective effect of vitamin D against respiratory infections, observational studies have also shown an association between low vitamin D status and adverse respiratory outcomes; these results do not necessarily prove a causal relationship. Furthermore, results from clinical trials investigating the effects of vitamin D supplementation have been mixed [57, 58], with some showing no significant effect on respiratory infections [59]. Therefore, the potential protective benefits of vitamin D identified in mechanistic and observational studies cannot be directly translated into clinical benefits of supplementation.

Overall, vitamin D exerts multiple effects on the immune system by modulating innate and adaptive immunity, regulating the production of antimicrobial peptides, maintaining epithelial barrier integrity, and regulating inflammatory responses. These immunomodulatory properties may contribute to antiviral immune responses, protection against viral respiratory infections and reduction of inflammation‐mediated tissue damage. Finally, the modulatory effects of vitamin D extend beyond the host immune system and involve complex bidirectional interactions with the microbiota.

## 5. Microbiota as a Key Modulator

The microbiota is a dynamic and complex population of microorganisms that settles in different parts of the body from birth [40]. The majority of microbiota are located in the gut, but they also exist in other parts of the body, such as the skin, reproductive system, and lungs [4, 49]. The dominant bacterial phyla in the lungs are similar to the gut, mainly Firmicutes and Bacteroidetes, followed by Proteobacteria and Actinobacteria [60].

Microbiota play a role in health and disease [57]. Microbiota are placed on the mucous surfaces of the body and prevent the establishment of pathogens and the overgrowth of pathogens through pathogen colonization resistance [58]. Microbiota are involved in energy metabolism and the production of vitamins B and K [61]. This dynamic ecosystem affects many immune responses and metabolic pathways in humans. Many immune‐mediated diseases, metabolic diseases, and cancers are associated with microbiota dysbiosis [5, 62].

Microbiota play a role in the development and training of the immune system, and from the time of birth, the microbiota and the immune system grow and evolve in parallel [63]. Microbiota metabolites can affect the differentiation and function of immune system cells [64]. Many of the receptors for these metabolites (SCFAs, tryptophan metabolites, and secondary bile acids) are located on immune system cells [65]. In general, microbiota metabolites have anti‐inflammatory properties and increase the differentiation and function of immunosuppressive cells [63]. They also inhibit inflammatory cells and thus contribute to immune homeostasis [64].

There is emerging evidence that the microbiota is also involved in the induction of trained immunity at mucosal surfaces [3]. Although adaptive immune memory is antigen‐specific, mediated by B and T lymphocytes, and is responsible for the long‐lasting immunological memory seen after infections, trained immunity is mediated through epigenetic and metabolic reprogramming of innate immune cells after exposure to microbial‐associated molecular patterns (MAMPs) such as lipopolysaccharide and other microbial components that are recognized by PRRs expressed on innate immune cells and stimulate intracellular signaling pathways that lead to metabolic changes and epigenetic remodeling, resulting in long‐lasting metabolic and epigenetic changes that increase the ability of innate immune cells to respond more quickly and robustly to subsequent microbial or viral challenges [3, 66]. Trained immunity plays a role in mucosal immune homeostasis and antiviral defense in barrier tissues.

Germ‐free mice have an underdeveloped immune system [67], which is evidence of the role of the microbiota in the formation of the immune system. Interferons are one of the important components of the immune system in the context of viral respiratory infections [68]. Interferons are the components of the innate immune system that control viral respiratory infections, and microbiotas are one of the main factors regulating interferon production and homeostasis of interferon responses [69]. Even without infection, there is always a basal level of interferon in the body, and the microbiota are the main factors in the production of interferons (presentation of PAMPs and activation of TLRs) in infection‐naive conditions, which ensures that in case of infection, antiviral responses are formed in time [69].

The microbiota influences both local and systemic immune responses [63]. The lung microbiota also influences local lung immunity and homeostasis [70]. Colonization of the microbiota in the lungs signals the maturation of lung immune cells [70, 71]. In addition to shaping the local tissue immune system, the microbiota influences the systemic immune system [60, 64]. Because their metabolites can enter the bloodstream and affect immune cells in distant locations [60]. Consequently, any factor affecting the composition of the microbiota results in alterations in immune responses to pathogens.

Although most studies have examined the relationship between vitamin D and the gut microbiota, these effects may extend to the lungs as well [72]. The influence of the gut microbiota on the respiratory system can be mediated by the gut–lung axis (GLA), through the bidirectional communication between the gut and the lungs, where the microbiota in each organ can influence the other and impact respiratory health [73]. Changes in the composition of the gut microbiota and their metabolites affect lung health and lung diseases [74], just as respiratory infections can alter the composition of the gut microbiota by inducing systemic inflammation, migration of immune cells, modulation of mucosal immunity, changes in gut barrier integrity and interestingly by inducing inappetence that changes eating habits then influences the gut microbiome and its metabolic processes [73, 75]. Communication along the GLA is done in several ways [12]: (i) transfer of microbiota between two organs, (ii) trafficking of immune cells between the two organs, and (iii) transfer of microbial metabolites and signaling molecules between the lungs and intestines; in this way, microbiota metabolites, including SCFAs, enter the blood circulation and can affect the immune system in distant areas (such as the lung). For example, SCFAs produced in the gut are involved in pulmonary defense responses. SCFAs, which are products of the fermentation of dietary fibers by the microbiota in the gut, play a pivotal role in regulating the immune system. SCFAs play an important role in controlling excessive inflammation in respiratory pathologies and viral respiratory infections, such as influenza and COVID‐19, by promoting regulatory T cell expansion, suppressing inflammatory T helper 17 cells, and restoring a Treg/Th17 balance [74]. In addition to bacterial body components, other microbiota metabolites and immune signals move between these two organs (lung and gut) through the bloodstream. The connection between the gut and the lungs, two anatomically separate organs, is regulated by blood flow and the lymphatic system [12, 76]. In fact, the intestinal microbiota and their metabolites affect pulmonary immunity through the GLA [360]. For example, animal studies have shown that antibiotic treatment in mice leads to a decrease in immune responses against influenza [60]. On the other hand, lung inflammation affects the intestinal microbiota and indicates the two‐way communication between the lung and intestine via the GLA [75, 77].

In fact, GLA is a dynamic dialogue between the lung and gut microbiota that shapes immune responses and affects the pathogenesis of respiratory diseases [60]. On the other hand, it seems that the lines of communication between the intestine and the lung (GLA) may increase due to the dysfunction of the epithelial barriers (both organs) in the elderly (enhanced GLA; in other words, the connection along the GLA increases with age) [12].

In many studies, the relationship between the microbiota and respiratory infections has been investigated [78]. One article discusses the connection between respiratory microbiota and the severity of COVID‐19 illness. The research indicated that COVID‐19 patients had a higher Firmicutes‐to‐Bacteroides ratio. A higher frequency of Firmicutes and a lower frequency of Bacteroidetes were observed in patients with severe and fatal COVID‐19 [78].

Changes in the frequency and composition of lung microbiota communities (lung microbiota dysbiosis) may lead to alterations in the host′s immune response, increased inflammation, and the development of lung damage [79]. Another study′s findings indicate that the lung microbiota is associated with the ARDS outcomes in COVID‐19 patients (either resolving or nonresolving) [80].

In other studies, the impacts of microbiota metabolites on viral respiratory infections (such as the protective effect of butyrate against influenza) have been observed. The entry of a large number of neutrophils or their long‐term persistence in the lung during influenza infection contributes to tissue damage [10]. Butyrate causes a decrease in the entry of neutrophils into the respiratory tract of mice infected with the influenza virus [81]. Acetate also has antiviral and protective effects against RSV infection by increasing beta‐interferon production [81]. Another metabolite of the microbiota is secondary bile acids. Secondary bile acids can enter the blood and intestines. They possibly can destroy the viral envelope or affect the cell membranes and may harm the attachment and entry of viruses into cells [11].

Overall, the microbiota acts as a key modulator of host immunity by shaping immune system development, training the immune system, maintaining immune homeostasis, regulating inflammatory responses, and mediating gut–lung communication. Through these mechanisms, microbiota communities and their metabolites may influence susceptibility to viral respiratory infections and disease severity. Age‐related changes in microbiota composition may disrupt trained immunity and affect antiviral immune responses.

## 6. Bidirectional Interaction Between Vitamin D/VDR and Microbiota

Scientific evidence suggests that the relationship between vitamin D/VDR and microbiota is bidirectional, playing an important role in mucosal immune homeostasis and antiviral defense. Microbiota affect vitamin D signaling pathways through their metabolites—for example, secondary bile acids act as ligands for VDR. Secondary bile acids, by activating VDR, lead to the production of antimicrobial peptides and the inhibition of pathogenic bacterial proliferation, followed by a shift in the microbiota towards a healthier composition [5]. Other microbiota metabolites, such as SCFAs (butyrate), also affect vitamin D signaling pathways and immune homeostasis by regulating the expression of the VDR receptor [82]. It also appears that microbiota may influence vitamin D absorption. Microbiota regulate pathways of lipid metabolism and fatty acid synthesis, which are involved in the absorption of vitamin D (a fat‐soluble vitamin) [83]. In addition, evidence from an animal study suggests that the microbiota also influence vitamin D metabolism [84]. There is also evidence that some bacteria express homologues of enzymes involved in vitamin D metabolism, such as CYP27B1, supporting the influence of the microbiota on vitamin D metabolism [85]. In contrast, vitamin D and VDR‐related signaling pathways play a role in maintaining mucosal homeostasis and shaping the microbiota by producing antimicrobial peptides, modulating immune responses, and maintaining epithelial barrier integrity [30]. Therefore, the bidirectional interaction between vitamin D/VDR and the microbiota may influence antiviral immune responses and the severity of respiratory viral infections in the elderly by regulating mucosal immune homeostasis and microbiota composition.

The interaction between vitamin D/VDR and the microbiota is mediated by a set of interconnected mechanisms 8]. This axis plays an important role in maintaining mucosal immune homeostasis and the balance between immune tolerance and protective responses. Vitamin D/VDR and microbiota‐derived metabolites act cooperatively to regulate the production of antimicrobial peptides, modulate innate and adaptive immune responses, maintain the integrity of epithelial barriers, and shape the mucosal immune environment [8]. VDR‐dependent signaling increases the expression of antimicrobial peptides and tight junction proteins, thereby preventing microbiota translocation and reducing mucosal inflammation [30]. On the other hand, microbiota‐derived metabolites, including SCFA and secondary bile acids, can help maintain immune homeostasis by modulating VDR signaling pathways and regulating the mucosal immune environment. Also, both vitamin D/VDR and the microbiota play a role in reducing inflammation and maintaining immune tolerance to the microbiota at mucosal surfaces by inducing T regulatory cells [30, 86].

Aging affects almost all components of the vitamin D–microbiota axis. Vitamin D deficiency is highly prevalent in the elderly, and aging is often associated with microbiota dysbiosis, impaired mucosal barrier function, and inflammaging. In addition, age‐related changes in immune function can disrupt the balance between immune tolerance and protective responses. Thus, aging may simultaneously affect both arms of the vitamin D/VDR and microbiota interaction, leading to impaired mucosal immune homeostasis, increased inflammation, and reduced efficacy of antiviral responses.

On the other hand, evidence shows that VDR knockout mice had a higher frequency of *Salmonella typhimurium* infection than the wild‐type group [5, 87]. Another study showed that VDR knockout mice had higher levels of *Clostridium* and lower levels of *Lactobacillus* compared with the control group [5]. Animal studies have reported that mice with a knockout of the Cyp27B1 gene and mice fed a low‐vitamin D diet show an increased abundance of Bacteroidetes in the gut microbiota [5].

Vitamin D deficiency in humans is associated with changes in the microbiota and inflammatory markers [5]. The results of a study in older men show that higher vitamin D levels are associated with a greater abundance of butyrate‐producing bacteria (a group of beneficial bacteria) [88]. There is also evidence on the effects of vitamin D supplementation on the human gut microbiota. A randomized clinical trial examined the effect of vitamin D supplementation on the fecal microbiota in 26 healthy adults with vitamin D deficiency. The study findings showed that the vitamin D group had a higher abundance of the genus *Lachnospira* and a lower abundance of the genus *Blautia* [89]. In a randomized, double‐blind, dose‐response study in healthy adults, the results showed that an increase in serum 25(OH)D following vitamin D supplementation was associated with increases in beneficial bacteria and decreases in pathogenic bacteria [90]. A systematic review of RCTs found that vitamin D supplementation was associated with a significant increase in the abundance of bacterial species with anti‐inflammatory and gut health–promoting properties, such as *Bifidobacterium* and *Lactobacillus* [91]. Overall, vitamin D may help create an anti‐inflammatory environment by modulating the gut microbiota, including increasing *Bifidobacterium* and *Lactobacillus*. These changes could influence the regulation of immune responses and possibly the severity of viral respiratory infections through the GLA.

Although most studies on vitamin D/VDR–microbiota have focused on the gut microbiota, this interaction may also apply to the respiratory microbiota. There are limited studies on the effect of vitamin D on the respiratory microbiota. However, one study reported that 12 weeks of vitamin D supplementation in cystic fibrosis patients affects the composition of the intestinal and airway microbiota and increases the frequency of beneficial microbiota [72]. Another study of 1005 infants hospitalized for bronchitis found an association between serum D levels and nasopharyngeal microbiota composition [92]. Although this initial finding suggests a relationship between vitamin D and respiratory microbiota, further studies are needed to investigate the impact of the vitamin D/VDR–respiratory microbiota axis on the severity of viral respiratory infections in the elderly.

**Figure 2:**
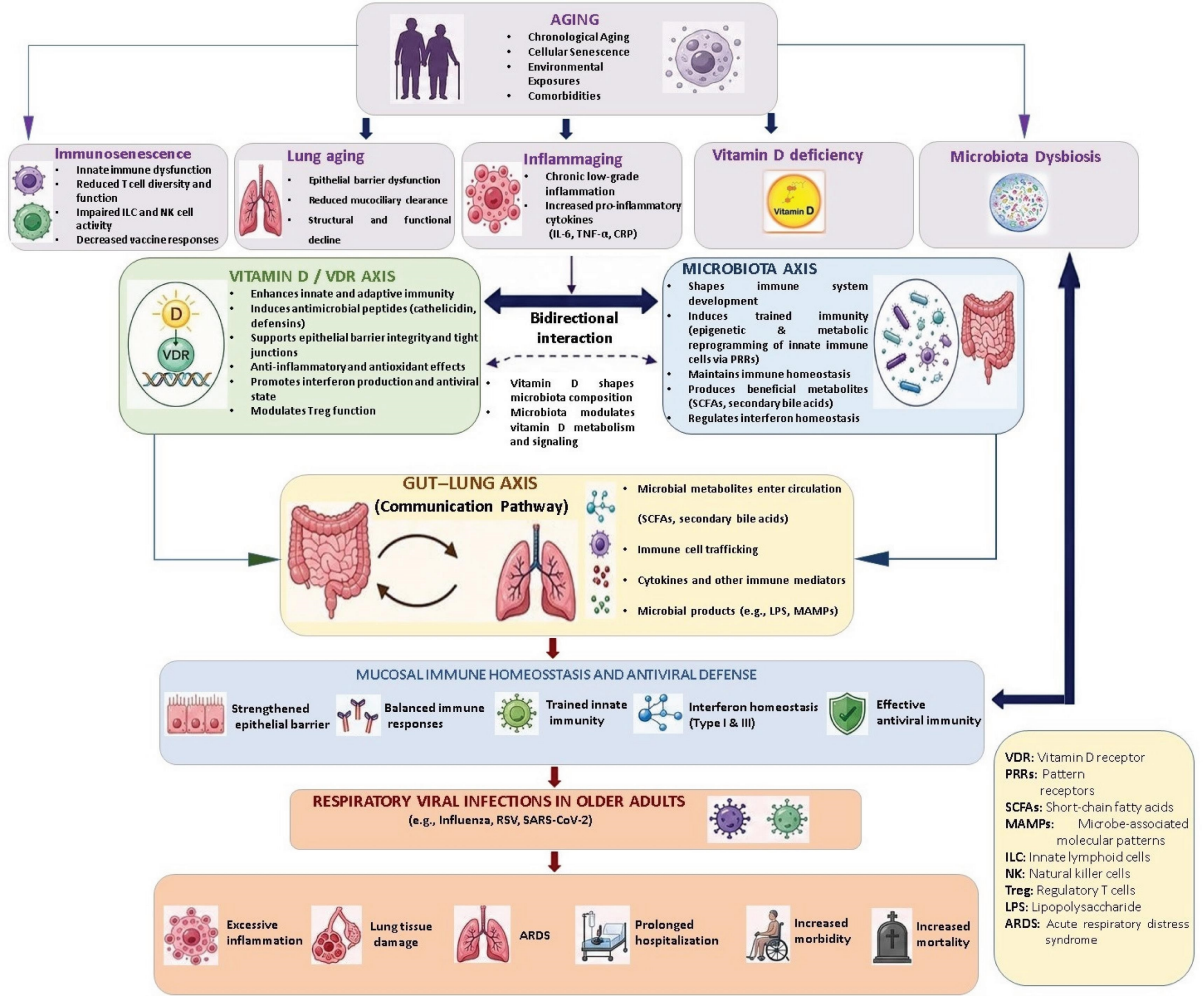
The aging–vitamin D/VDR–microbiota axis and its potential role in mucosal immune homeostasis and antiviral defense. The bidirectional arrows represent reciprocal interactions between vitamin D/VDR signaling and the microbiota. Microbiota‐derived metabolites, particularly short‐chain fatty acids (SCFAs) and secondary bile acids, can modulate VDR signaling. Vitamin D/VDR activation can shape microbiota composition and function through antimicrobial peptide production, epithelial barrier maintenance, and immune regulation. These interconnected pathways with the GLA provide an important route for communication between intestinal and respiratory compartments and may contribute to mucosal immune homeostasis and antiviral defense.

Overall, the vitamin D/VDR–microbiota axis can be considered as a complex regulatory network that plays an important role in maintaining immune homeostasis and host resistance to respiratory viral infections by regulating immune responses, maintaining mucosal barrier integrity, and shaping microbiota composition. Consequently, age‐related vitamin D deficiency and microbiota dysbiosis may act synergistically to impair mucosal immune homeostasis and increase susceptibility to respiratory viral infection (Figure 2).

## 7. Synthesis and Clinical Implications

Aging is associated with a series of physiological, immunological, and metabolic changes, disruption of epithelial barriers, vitamin D deficiency, and microbiota dysbiosis [2, 7, 10, 12, 40]. All of these factors contribute to increased susceptibility and vulnerability to viral infections. These factors may interact within an interconnected network, exerting a synergistic effect on the severity of respiratory infections in the elderly.

There is evidence that the permeability of the intestinal epithelium increases with age. A hypothesis called the enhanced GLA has been proposed in aging [12]. This hypothesis suggests that the tight junctions of these barriers are disrupted with age (both the mycobiota and vitamin D play a role in maintaining the integrity of the epithelial barrier by increasing the expression of tight junction proteins). These structures become leakier with age. A disrupted epithelial barrier may allow greater translocation of microbes and microbial metabolites, thereby enhancing pathological communication along the GLA in older individuals [12]. In this situation, changes in the gut microbiota could have more pronounced effects on mucosal and systemic respiratory immunity and thereby influence antiviral immunity and the severity of respiratory viral infections in older individuals.

Elderly individuals often experience vitamin D deficiency [6] and microbiota dysbiosis [7], which can be considered two modifiable host factors with potential for translation into clinical applications. Given the high prevalence of vitamin D deficiency in the elderly and its role in regulating immune responses, assessing vitamin D status and considering appropriate strategies to correct its deficiency may be clinically important in the elderly. Similarly, since aging is associated with changes in microbiota composition, targeted microbiota interventions aimed at restoring microbial homeostasis (such as probiotics, prebiotics, postbiotics, and synbiotics) may be considered as potential preventive or adjunctive strategies in the future. Although there is currently no conclusive evidence on the effect of correcting vitamin D status or microbiota‐based interventions on reducing the severity of respiratory viral infections in the elderly, appropriately designed clinical and translational studies are necessary to investigate the possibility of translating these biological insights into clinical applications.

Further studies are needed to better understand the interrelationships among aging, vitamin D, and the microbiota, and how their interactions affect antiviral immunity and the severity of respiratory viral infections. Longitudinal studies and prospective clinical trials can help clarify the causal relationships between vitamin D status, microbiota dysbiosis, and clinical outcomes of respiratory infections in the elderly.

Furthermore, most of the existing studies have focused on the gut microbiota, and the role of the lower respiratory tract microbiota in the elderly is still poorly understood. Investigating the interaction between vitamin D and the respiratory microbiota could provide new insights into the pathogenesis of respiratory viral infections in the elderly. Future studies should also evaluate the feasibility of vitamin D and microbiota‐based interventions, especially in combination, and their impact on immune responses and clinical outcomes of respiratory viral infections. The use of multiomics approaches and translational studies could also help identify molecular mechanisms and potential therapeutic targets in the aging–vitamin D–microbiota axis.

## 8. Conclusion and Future Directions

Overall, aging, vitamin D deficiency, and microbiota dysbiosis interact in a complex network that can synergistically impair mucosal immune homeostasis, thereby potentially affecting antiviral responses. Vitamin D deficiency is associated with changes in the gut microbial ecosystem, and similar interactions may occur in respiratory microbiota. As the evidence above shows, the higher frequency of vitamin D deficiency and the decrease in the diversity of the intestinal microbiota with age are important contributors to the increased susceptibility and severity of respiratory infections in older adults. In addition to serum vitamin D levels, changes in its metabolic pathways and bioavailability due to VDR gene polymorphisms also contribute to its effects on respiratory infections.

From a clinical perspective, it is important to screen for vitamin D deficiency in high‐risk elderly patients with recurrent respiratory infections. Although the optimal level of vitamin D for immune function is debated, correction of severe deficiency is recommended, especially in elderly patients with recurrent respiratory infections.

Although clinical trials on the effect of vitamin D supplementation on microbiota composition indicate that it can modulate the human microbiota, the available evidence has important limitations. Due to the heterogeneity of studies (differences in study populations, baseline vitamin D status, supplementation regimens, follow‐up duration, and methods of microbiota analysis), it is still not possible to state with certainty the cause‐and‐effect relationship and its clinical significance. There is a need to design more rigorous clinical trials that use standard methods for microbiota analysis. Future studies should prioritize determining whether vitamin D supplementation can beneficially alter the respiratory microbiota of older adults, clarify the role of VDR polymorphisms in susceptibility to respiratory infections through microbiota modulation, and evaluate the role of combination interventions, including vitamin D supplementation with prebiotics, probiotics, or synbiotics, on viral respiratory infections in well‐designed randomized clinical trials.

In the future, studies on viral respiratory infections in the elderly should focus more on the aging–vitamin D–microbiota axis rather than on the independent roles of vitamin D and the microbiota. A better understanding of this axis and its integration could help design personalized strategies for the prevention and management of respiratory diseases in the elderly. Finally, vitamin D and microbiota are modifiable targets, and a thorough understanding of their effects may contribute to better management of respiratory infections. In addition, the enhanced GLA hypothesis can serve as a conceptual framework to guide future research in this area.

## Funding

No funding was received for this manuscript.

## Conflicts of Interest

The authors declare no conflicts of interest.

## Data Availability

Data sharing is not applicable to this article as no datasets were generated or analysed during the current study.
